# Validity of Body Composition Estimates in Women Assessed by a Multifrequency Bioelectrical Impedance Device

**DOI:** 10.3390/s25165037

**Published:** 2025-08-14

**Authors:** Mitchell E. Zaplatosch, Juliana F. Meireles, Janeen S. Amason, Sadaf Dabeer, Brian M. Kliszczewicz, Gerald T. Mangine, Valene G. Barry, Barbara A. Gower, Katherine H. Ingram

**Affiliations:** 1 Department of Exercise Science and Sport Management, Kennesaw State University, Kennesaw, GA 30144, USA; mzaplato@kennesaw.edu (M.E.Z.);; 2 Department of Family and Community Medicine, School of Community Medicine, University of Oklahoma Health Sciences Center, Tulsa, OK 74135, USA; 3 Wellstar School of Nursing, Kennesaw State University, Kennesaw, GA 30144, USA; 4 Division of Endocrinology, Metabolism and Lipids, School of Medicine, Emory University, Atlanta, GA 30322, USA; 5 Department of Obstetrics and Gynecology, School of Medicine, Washington University in Saint Louis, St. Louis, MO 63110, USA; 6 Department of Nutrition Sciences, University of Alabama at Birmingham, Birmingham, AL 35233, USA

**Keywords:** total body water, bio-impedance, fat mass, lean mass, D2O, 4-compartment model, body composition

## Abstract

Background: Multifrequency bioelectrical impedance devices such as the InBody 770 (IB770) offer faster measurements and lower costs compared with other body composition assessments. This study validated measures from IB770 against the deuterium oxide dilution technique (D2O) and DXA and compared a four-compartment (4C) model using total body water (TBW) derived from IB770 compared with D2O. Methods: A total of 55 adult females (mean ± SD, age: 21.1 ± 2.6 years) completed IB770 and DXA scans and the D2O protocol. Lin’s concordance correlation coefficients (CCCs), Bland–Altman analyses, and other equivalence tests evaluated agreement between IB770 and the criterion for measures of fat mass (FM), fat-free mass (FFM), and TBW individually and as part of 4C models. Results: There was substantial agreement between IB770 and D2O for TBW (MD = Mean Difference) (MD = 0.34 L, CCC = 0.98) and between the IB770 and DXA for FM (MD = −0.22 kg, CCC = 0.99). IB770 overestimated FFM compared with DXA (MD = 3.15 kg, CCC = 0.91). Both 4C models had almost perfect agreement for FM (CCC = 0.99), FFM (CCC = 0.99), and body fat percentage (CCC = 0.99). Conclusions: IB770 is valid for assessing TBW and can be used within the context of a 4C model in females.

## 1. Introduction

Body composition metrics are used widely in research and clinical settings for monitoring the efficacy of lifestyle interventions and assessing risk for chronic disease. An extensive body of literature has focused on the development and validation of body composition assessments [1,2]. The “gold standard” method for estimating fat mass (FM) and fat-free mass (FFM) is the four-compartment (4C) model, a formula derived from a combination of several factors, including body mass, bone mineral density, body density, and body water [3]. Most of these factors require specialized equipment or additional time, making this model inaccessible or infeasible to many scientists, clinicians, and healthcare workers.

Among these techniques, dual-energy X-ray absorptiometry (DXA) is commonly used as a reference criterion for bone mineral density [4], fat mass (FM) [5], lean soft tissue [6], and can also be used to derive body volume [7]. Meanwhile, the deuterium oxide dilution method (D2O) is considered the “gold standard” for estimating total body water (TBW) in humans [8]. However, these methods have practical limitations. For instance, the use of X-rays by DXA to distinguish body compartments may be contraindicated for certain populations, including individuals who are pregnant or are undergoing cancer treatment. The D2O protocol requires consuming a deuterium oxide solution and waiting motionless for 4–6 h to allow body water compartments to equilibrate. This equilibration time limits the usefulness of this method in clinical or research settings with limited time to assess participants.

Bioelectrical impedance analysis (BIA) does not require consuming a tracer substance or exposure to radiation and is therefore an attractive, time-efficient alternative to D2O and DXA [9]. BIA estimates TBW, FM, and fat-free mass (FFM) by measuring the resistance and reactance of an electric current that passes through the intracellular and extracellular fluids of the body within 30–60 s. The method is based on the principle that the higher relative fluid composition of FFM contributes to greater electrical conductivity as opposed to the lower fluid content found within FM [10]. Single-frequency BIA devices measure the resistance at 50 kHz and are prone to error since this frequency cannot penetrate tissue completely to measure the entire muscle cell volume [11]. In contrast, multifrequency BIA analyzers use several frequencies, including higher frequencies (up to 1000 kHz) capable of penetrating cell membranes to estimate intracellular water content, and lower frequencies to estimate extracellular water. By accounting for differences in fluid distribution, multifrequency BIA devices provide estimates that are more accurate and consistent than those of single-frequency analyzers [12].

Few techniques are available to adequately assess body composition in pregnant individuals, and this can be limiting for research in maternal health. One major obstacle is that pregnancy is characterized by rapidly changing levels of fat and water stores [13]. The 4C model can account for fluctuations in total body water (TBW); however, it requires bone mineral content (BMC) via DXA and is, therefore, contraindicated during pregnancy because of potential radiation exposure. While it is possible to assess BMC after birth and compute body composition retroactively, this can only be accurate if BMC does not vary during gestation. BIA may be an attractive alternative for body composition assessment in this population because of its ability to assess body water content. However, Brewer et al. observed higher error in females using BIA compared with a 4C model [14], warranting further investigation in this population.

The underlying regression equations used to predict body composition by bioelectrical impedance rely upon an accurate estimation of TBW by the analyzer. Specifically, bioelectrical impedance calculations assume a stable ratio between TBW and FFM of approximately 0.73 [15]. A recent investigation by Blue et al. validated a specific multifrequency bioelectrical impedance device, the InBody 770 (IB770, Cerritos, CA, USA), against a four-compartment model using D2O obtained from saliva, finding excellent to very good agreement, which was consistent across race and ethnicity [16]. Similarly, Brewer et al. concluded overall good agreement between the estimated FM and FFM obtained from the IB770 compared with a 4C model, suggesting the device might be more practical and reasonably accurate for assessing body composition [14]. However, Brewer also noted that IB770 significantly overestimated body fat percentage and fat mass, while underestimating fat-free mass compared with a 4C model. Neither of these investigations explicitly assessed the validity of using TBW values derived from IB770 within a 4C model. If a 4C model incorporating results from IB770 displays similar agreement with the traditional 4C model, this could save investigators both the time and expense of the deuterium dilution method for TBW measurements.

The purpose of our study was to assess the validity of the IB770 for estimating TBW, fat-mass, and fat-free mass compared with urinary D2O and DXA, using a rigorous panel of statistical equivalence measures. We also assessed the validity of a 4C model using TBW estimates derived from IB770 compared with a 4C model using D2O to estimate TBW. We predicted strong agreement between the IB770 and D2O for TBW assessment and an underestimation of FM and overestimation of FFM by IB770 when compared with DXA, as observed in previous studies. In the context of a 4C model, we predicted body composition estimates would be similar when using TBW derived from IB770 in this model compared with D2O.

## 2. Materials and Methods

### 2.1. Participants

Fifty-nine apparently healthy women aged 18–29 were recruited from Kennesaw State University (KSU) and the local community and screened over the phone prior to enrollment in the study. Individuals who were pregnant or had either diabetes or other conditions or medication known to affect metabolism were excluded from the study. Four of these participants were excluded from the final analyses for having missing IB770 data (n = 3) or exceeding the limits of the DXA table (n = 1). Thus, the final cohort included 55 women (n = 22 (40%) Non-Hispanic Black, n = 21 (38.2%) Non-Hispanic White, n = 5 (9.1%) Hispanic, n = 6 (10.9%) Asian, and n = 1 (1.8%) other), aged 21.1 ± 2.6 years, and with a mean body mass index (BMI) of 25.8 ± 5.5 kg/m^2^. Participants were not scheduled based on menstrual cycle phase, with 41% reporting the use of some form of contraceptive. Each participant confirmed that she was not pregnant prior to testing and was offered a pregnancy test. The Institutional Review Board of KSU approved these protocols, and all participants provided verbal and written consent prior to data collection (KSU IRB #18-346).

### 2.2. Experimental Design

**Figure 1:**
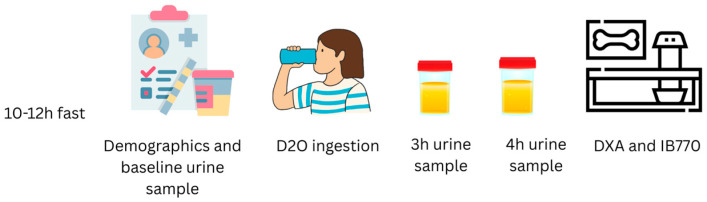
Study timeline. DXA image from: <a href=https://www.flaticon.com/free-icons/dexa-scan title=dexa scan icons”>Dexa scan icons created by Freepik – Flaticon</a>, accessed on 5 June 2025.

Participants made one visit to the KSU Exercise Science Physiology Laboratory in the morning following an overnight fast, in which they were instructed to ingest nothing by mouth, including water, for 10–12 h (Figure 1). Participants were instructed to wear light, metal-free clothing and were asked to remove shoes, jewelry, and any other metal prior to body weight and composition assessments. Height (±0.01 m) and weight (±0.01 kg) were collected using a stadiometer with a digital scale (WB-3000, Tanita Corporation, Arlington Heights, IL, USA).

### 2.3. Total Body Water by Deuterium Oxide Dilution Technique (D2O)

Participants provided baseline urine samples before drinking a 10 g deuterium oxide solution (Cambridge Laboratories), then reclined comfortably with minimal movement until urine samples were collected at 3 and 4 h post-consumption. Urine samples were stored at 0 °C until they were shipped to the Metabolism Core Laboratory at the University of Alabama at Birmingham for TBW assessment via isotope ratio mass spectrometry, as previously described (8). The dilution space of deuterium is calculated using the following equation:

TBW(moles)=[(WA×(Δdose−Δtap))/(18.02×a(Δpost−Δpre))]

In this equation, W = Amount of water (grams) used to dilute the dose, A = Amount of dose (grams) administered to subject, a = Amount of dose (grams) diluted for analysis, and Δ = the delta value from the isotope ratio mass spectrometry.

Total body water (N) is calculated with Equation 2 from Speakman [17]:

$$N=((N_{0}/1.007)\;+\;(N_{d}/1.043))/2$$

In this equation, N_0_ represents the amount of water contained within the body at the reference dilution space, and N_d_ represents the amount of water contained within the body at the dilution space of interest.

### 2.4. Bioelectrical Impedance Analysis

Body composition data were collected using the IB770 (InBody^®^ 770; InBodyUSA, Cerritos, CA, USA), according to the manufacturer’s instructions, following the final urine sample from the D2O measurements. Prior to measurement, each participant was instructed to void her bladder. Feet and palms were cleaned of debris using manufacturer-supplied tissue wipes containing 0.9% NaCl. Demographic information was entered when prompted, and the participant stood motionless as instructed while holding sensors with arms lifted away from the trunk at approximately a 45° angle, per manufacturer guidelines. The IB770 collected impedance data at six frequencies: 1 kHz, 5 kHz, 50 kHz, 250 kHz, 500 kHz, and 1000 kHz. Each scan lasted approximately 1–2 min and provided total body estimates of FM, FFM, and TBW.

### 2.5. Dual-Energy X-Ray Absorptiometry (DXA)

Daily calibrations were completed for DXA (GE Lunar Prodigy, Software version 13.60, GE Lunar Corp., Madison, WI, USA) according to manufacturer instructions. Body composition data were collected with the participant lying supine and motionless throughout the 6 to 10 min scan. The arms were positioned flat against the table, while knees and ankles were stabilized using manufacturer-provided Velcro straps. A trained staff member confirmed accurate regions of interest per the manufacturer’s specifications. The DXA scan provided total FM and FFM.

### 2.6. Fat-Free Mass Hydration

For descriptive purposes, fat-free mass hydration was calculated for each participant by dividing TBW by the estimate of FFM derived from each 4C model [15].

### 2.7. Four-Compartment Model

Two four-compartment models were used to estimate body fat, percent body fat, and fat-free mass according to the equation of Wang et al. [18].

$$\begin{array}{l} FM\left(kg\right)=2.748\left(BV\right)-0.699\left(TBW\right)+1.129\left(Mo\right)-2.051\left(BM\right);\\ \%BF=\left(\frac{FM}{BM}\right)\times 100;\\ FFM\left(kg\right)=BM-FM \end{array}$$

In this equation, total body mineral density (Mo) was obtained from DXA-measured bone mineral content (BMC) [14], where:

Mo = BMC × 1.0436

Body volume (BV) was obtained from the DXA-derived value from a previously validated prediction equation created by Smith-Ryan et al. [7]:

$$DEXA\;Volume\left(L\right)=\frac{FM}{0.84}+\frac{LM}{1.03}+\frac{BMC}{11.63}+(-3.12)$$

TBW in either the four-compartment model was derived from either IB770 or the D2O method to obtain two sets of four-compartment measurements for comparison.

### 2.8. Statistical Analysis

Estimates of FM, FFM, and BF% were compared between both 4C models, and between DXA and IB770 for FM and FFM, and D2O and IB770 for TBW. Comparisons were completed using Lin’s concordance correlation coefficient (CCC) [19]. Agreement cutoffs for CCC have previously been defined as: poor (<0.90), moderate (0.90–0.95), substantial (0.95–0.99), and almost perfect (>0.99). The two one-sided *t*-test (TOST) [20] was performed with a 5% equivalence test of agreement between estimates from the IB770 and each criterion measure for FM, FFM, and TBW. This analysis involves specifying an upper and lower bound such that results falling within this range represent the absence of a significant difference. The TOST technique tests two null hypotheses: (1) that the difference between measures is greater than the upper equivalence bound, and (2) that the difference between measures is lower than the lower bound equivalence bound, with the alternative hypotheses suggesting the mean difference lies between each boundary and is close enough to zero for the measures to be practically equivalent [20]. The acceptable threshold for the TOST test between the IB770 and DXA was set at 1 kg, consistent with a previous investigation in children [21]. A 1 L equivalence region for TBW was selected based on a prior investigation assessing agreement between a different bioelectrical impedance device and D2O [22]. Additional agreement measures comparing the D2O derived 4C model and the IB770 4C model body composition measurements included constant error (CE = actual (4C model with D2O)–predicted (4C model with IB770), root mean square error (RMSE = √∑[predicted-actual]^2^/n), and standard error of the estimate (SEE = √∑[predicted-actual] × √1 − r^2^) for %BF, FM, and FFM. Agreement between corresponding measures was assessed using Bland and Altman’s 95% limits of agreement (LOA) technique (mean bias ± [1.96 × SD of differences]). The normality of the residuals was assessed visually via normal quantile plots and with the Shapiro–Wilk test [23]. The homogeneity of variance was assessed visually and with the Breusch–Pagan test [24]. All Bland–Altman analyses were assessed for proportional bias by regressing the difference between devices on the mean of both devices [25]. Analyses were performed using SPSS (v.27.0, Chicago, IL, USA) and R Studio (31) (4.3.1) using the “SimplyAgree” [26] and “TOSTER” package [20] with statistical significance set at *p* < 0.05.

## 3. Results

### 3.1. Total Body Water

**Figure 2:**
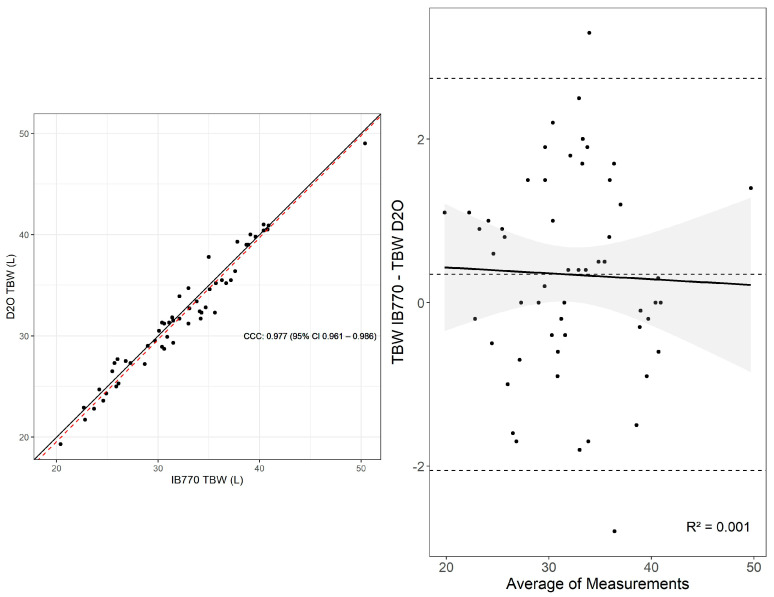
Lin’s concordance correlation (CCC) and Bland–Altman plot for TBW assessed via IB770 and D2O. Upper and lower dashed lines in the Bland-Altman plot represent the upper and lower bounds for the 95% limits of agreement between measures, with the middle dashed line representing the mean difference between measures.

The equivalence test between TBW estimates by the D2O and IB770 was significant, t(54) = 3.98, *p* < 0.01. However, the null hypothesis test was also significant, t(54) = 2.08, *p* = 0.04. Thus, the estimates were significantly different, though the magnitude of this difference was small enough to qualify as equivalent within the 1 L limits specified (Table 1). Similarly, Lin’s concordance correlation coefficient showed substantial agreement between measurements, similar to the Bland–Altman limits of agreement (Mean Difference (MD) [95% C.I.] = 0.34 L [0.01, 0.67], Lower LOA [95% C.I.] = −2.06 L [−2.53, −1.58], Upper LOA [95% C.I.] = 2.74 L [2.27, 3.22] (Figure 2). No proportional bias was detected with greater mean TBW (*p* = 0.803, Figure 2). Additional statistics supported good agreement between measures (CE = 0.34 L, RMSE = 1.21 L, SEE: 1.23 L).

### 3.2. Fat Mass

**Figure 3:**
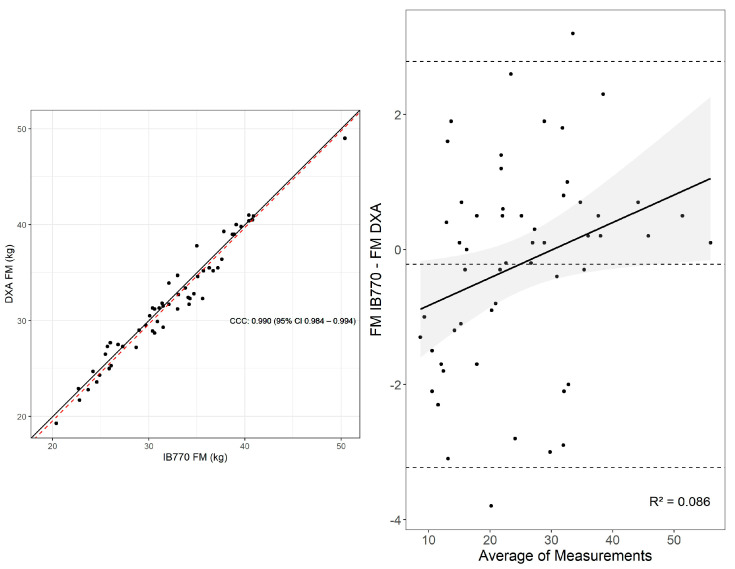
Lin’s concordance correlation (CCC) and Bland–Altman plot for FM assessed via IB770 and DXA. Upper and lower dashed lines in the Bland-Altman plot represent the upper and lower bounds for the 95% limits of agreement between measures, with the middle dashed line representing the mean difference between measures.

The equivalence test between estimates of FM by D2O and IB770 was significant, t(54) = 3.76, *p* < 0.01. The null hypothesis test was not significant (t(54) = −1.07, *p* = 0.29). Thus, the measures were not significantly different and were statistically equivalent within the 1 kg limits specified (Table 1). Similarly, Lin’s concordance correlation coefficient showed substantial agreement between measurements (Figure 3), similar to the Bland–Altman mean bias and limits of agreement (MD [95% C.I.] = −0.22 kg [−0.64, 0.19], Lower LOA [95% C.I.] = −3.23 kg [−3.82, −2.64], Upper LOA [95% C.I.] = 2.79 kg [2.19, 3.38]) (Figure 3). However, significant proportional bias was observed for FM assessment (*p* = 0.029), with an increasing difference between estimates as mean FM increased (Figure 1). Adjusting for proportional bias yielded similar results (MD [95% C.I.]: −0.22 kg [−0.62, 0.18], Lower LOA [95% C.I.] = −3.12 kg [−3.70, −2.55], Upper LOA [95% C.I.] = 2.68 kg [2.10, 3.25]. Additional statistics supported good agreement between measures (CE = −0.22 kg, RMSE = 1.42 kg, SEE = 1.45 kg).

### 3.3. Fat-Free Mass

**Figure 4:**
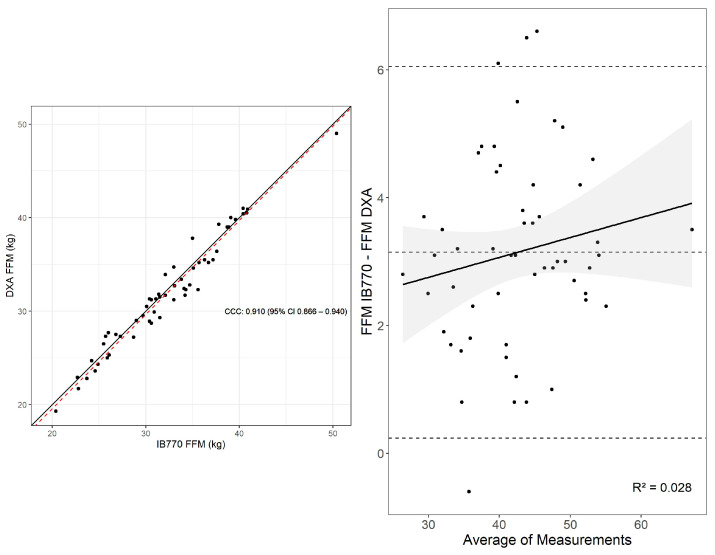
Lin’s concordance correlation (CCC) and Bland–Altman plot for FFM assessed via IB770 and DXA. Upper and lower dashed lines in the Bland-Altman plot represent the upper and lower bounds for the 95% limits of agreement between measures, with the middle dashed line representing the mean difference between measures.

The equivalence test for FFM between DXA and IB770 was not significant (t(54) = 10.73, *p* = 0.999), as estimates did not conform to the specified 1 kg limits. The null hypothesis test was significant, t(54) = 15.7, *p* < 0.01. Thus, the estimates of FFM were not statistically equivalent, with significantly higher FFM indicated by the IB770 assessment (Figure 3). Similarly, Lin’s concordance correlation was lower than the previous comparisons, and more observations lay outside the LOA in Bland–Altman analyses (MD [95% C.I.] = 3.15 kg [2.74, 3.54], Lower LOA [95% C.I.] = 0.24 kg [−0.33, 0.82], Upper LOA [95% C.I.] = 6.05 kg [5.48, 6.63] (Figure 4). No proportional bias was observed between measurements (*p* = 0.225). Additional statistics supported less agreement between measures (CE = 3.15 kg, RMSE = 1.42 kg, SEE = 1.45 kg).

### 3.4. Four-Compartment Model with Inbody 770 vs. D2O

**Figure 5:**
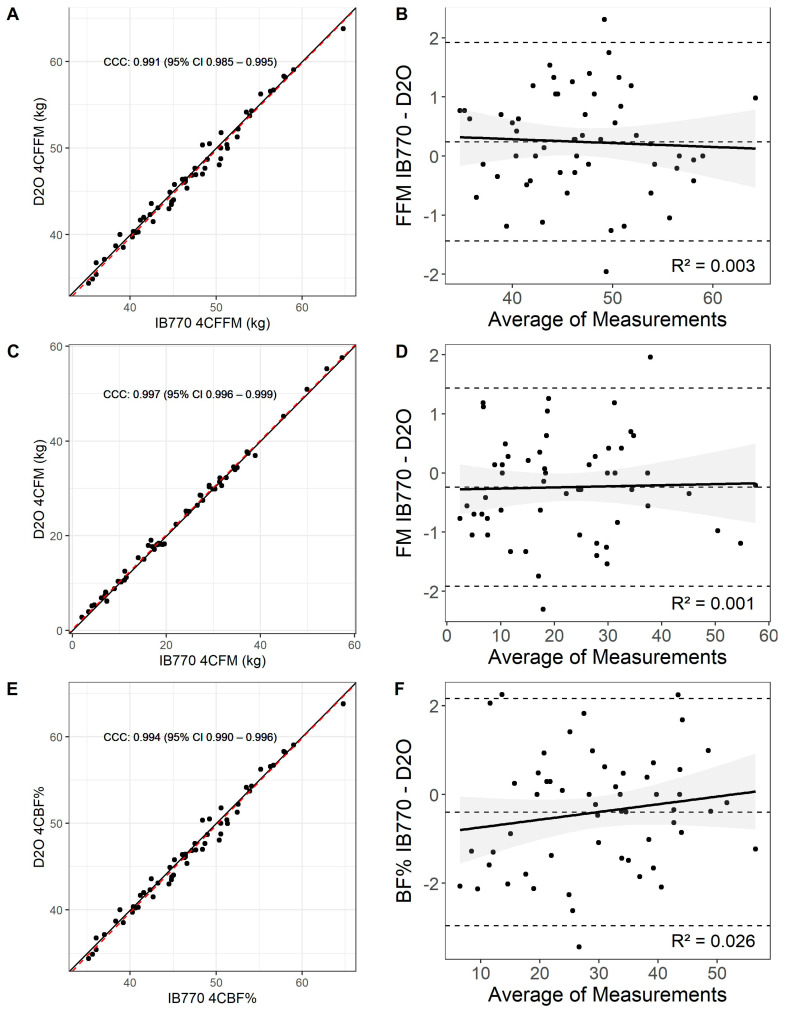
Lin’s concordance correlation (CCC) (**A**,**C**,**E**) and Bland–Altman plot (**B**,**D**,**F**) for 4C models using D2O or IB770 estimates of TBW. Upper and lower dashed lines in the Bland-Altman plots represent the upper and lower bounds for the 95% limits of agreement between measures, with the middle dashed line representing the mean difference between measures.

There was almost perfect agreement for FFM (CCC = 0.991 [0.985, 0.9948], CE = 0.240, RMSE = 0.848, SEE = 0.864) obtained from a 4C model using TBW estimated from the D2O method compared with a 4C model using TBW estimated from IB770. Most observations were within the limits of agreement (MD [95% C.I.] = 0.24 kg [0.01, 0.47], Lower LOA [95% C.I.] = −1.43 kg [−1.769, −1.105], Upper LOA = 1.918 kg [1.586, 2.250] with no significant proportional bias (*p* = 0.713) (Figure 5).

Similarly, there was an almost perfect agreement for FM (CCC = 0.997 [0.996, 0.999], CE = −0.240, RMSE = 0.846, SEE = 0.862) obtained from a 4C model using TBW estimated from the D2O method compared with a 4C model using TBW estimated from IB770. Most observations were within the limits of agreement (MD [95% C.I.] = −0.24 kg [−0.47, −0.01], Lower LOA [95% C.I.] = −1.92 [−2.25, −1.59], Upper LOA [95% C.I.] = 1.44 [1.105, 1.77], with no significant proportional bias (*p* = 0.834) (Figure 5).

Body fat percentage had almost perfect agreement between 4C model methods (CCC = 0.994, [0.990, 0.996], CE = −0.398%, RMSE = 1.264%, SEE = 1.288%). Most observations were within the limits of agreement (MD [95% C.I.] = −0.40% [−0.75, −0.05], Lower LOA [95% C.I.] = −2.96% [−3.47, −2.45] Upper LOA [95% C.I.] = 2.16% [1.66, 2.67]) with no significant proportional bias (*p* = 0.235) (Figure 5).

TOST results suggested FFM was practically equivalent (t(54) = 6.58, *p* < 0.01), although the null hypothesis significance test indicated significant differences (t(54) = −2.08, *p* = 0.04). Effects were similar for FM, which was practically equivalent (t(5) = −6.6, *p* < 0.01), despite a significant null hypothesis significance test (t(54) = 2.81, *p* = 0.04). Body fat percentage, while statistically different based on the null hypothesis significance test (t(54) = 2.260, *p* = 0.028), was also within an equivalence bounds of 1% (t(54) = −3.4, *p* < 0.01).

## 4. Discussion

In this study, we assessed the agreement between IB770 and measures of total body water, fat mass, and fat-free mass in a racially diverse, female population characterized by a wide range of body sizes. We also assessed the validity of a 4C model of body composition incorporating TBW derived from IB770 compared with a model incorporating TBW estimated from D2O. Our data support the use of the IB770 as a suitable alternative for estimating TBW both in isolation and as part of a four-compartment model. However, the FM and FFM estimates produced by the IB770 were not in complete agreement with DXA, suggesting these methods should not be used interchangeably.

Our findings indicate that TBW estimates by IB770 and D2O are practically equivalent within the 1 L bounds set by the TOST procedure. The TOST procedure is inherently dependent upon equivalence boundaries defined by the researcher [26]. There is currently no well-defined standard for an equivalence region expected from TBW estimation; therefore, the range used for this study was selected to maintain consistency with the agreement observed in a previous study. It is possible that some scenarios may benefit from a lower equivalence bound. Estimates from IB770 and D2O had a near-perfect relationship using Lin’s correlation coefficients. These estimates further maintained consistent agreement across the range of values, according to the Bland–Altman analysis. Previous studies have reported good precision for TBW estimates from other BIA devices when compared with the D2O method [27,28,29]. In particular, the agreement between IB770 and D2O in our study aligns with the previously observed agreement from Blue et al. [16] and Cataldi et al., who each used salivary D2O as the reference criteria. We observed slightly lower error between measurements in our study (SEE = 1.23 L) compared with Blue et al. (SEE = 1.44 L), as well as a lower mean difference (0.34 L vs. 1.3 L) which may be attributed to our sample being entirely female and on average smaller than the mixed sex sample tested by Blue [16]. While D2O is widely considered the gold standard for in vivo assessment of TBW, this technique is impractical for most applications because of its time-consuming protocol, high cost, and required technical expertise. Overall, our findings indicate that TBW measurements from the IB770 provide a valid alternative for assessing TBW, particularly when studies are not concerned with deviations within the 1 L equivalence bounds tested in the present study. This was consistent when including TBW derived from IB770 as part of a 4C model, suggesting that laboratories interested in assessing body composition do not need to rely on the added expense and time involved in assessing total body water via the D2O method. This device may also be particularly well-suited for studies or clinics assessing TBW fluctuations. For example, pregnancy is characterized by a substantial 7–8 L retention in TBW over the course of gestation [30]. However, there have been mixed findings regarding the utility of bioelectrical impedance spectroscopy on body water assessment in this population [9,30,31]. Given that water accumulation during pregnancy, including FFM hydration, is quite variable [32], this may further complicate the use of such methods throughout pregnancy but warrants further investigation. To prevent the potential for unethical radiation exposure during pregnancy, future validation in pregnant females may be warranted against other valid assessments, such as MRI [33]. We did not assess the efficacy of this method on the distribution of TBW between extracellular and intracellular fluid compartments. We recommend that future research incorporate the bromide dilution method for the assessment of extracellular water and account for additional confounders such as postural shifts on body water distribution [34], particularly changing body positions between assessment methods, which would be relevant during pregnancy.

Our findings indicate that IB770 and DXA were not in agreement when measuring FM. Although the mean difference between devices was within our predetermined limits, our results demonstrated significant proportional bias as fat mass increased, which is consistent with previous research from our laboratory in a mixed male and female sample [35]. Interestingly, while the overall lower FM observed from IB770 was consistent with previous observations, some participants with higher body mass had a higher FM value using the IB770 when compared with DXA (Figure 2). Thus, there is some inter-individual variability in the accuracy of the IB770 between participants. Total FFM was overestimated by IB770, as compared with the DXA criterion measure in our study, in agreement with McLester et al. [35] and others [36,37,38,39]. This difference is likely related to the inherent assumption underlying the bioelectrical impedance, assuming a stable ratio of 0.73 between TBW and FFM [15]. This assumption may not be true in certain individuals, particularly those who have a higher body fat percentage [40,41]. In fact, Tinsley et al. demonstrated that lean soft tissue hydration was a significant predictor of discrepancies between DXA-derived estimates of FM and FFM [42]. Thus, if researchers are relying on the IB770 as an accurate measurement of FFM, care should be taken to either ensure euhydration of participants or include a measure of hydration as a covariate in the analyses [43]. Additionally, increases in glycogen content within FFM can further alter this relationship as a result of increased stored intracellular water [44]. In our sample, estimates of FFM hydration in each 4C model were lower than expected (0.68 ± 0.05 and 0.68 ± 0.04 for D2O and IB770 4C models, respectively). Thus, it is possible that the FFM overestimation from the IB770 is due in part to the lower than expected FFM hydration in our sample. While we were unable to assess hydration status in the present study, we suggest future investigators completing similar investigations relying upon the IB770 to estimate FFM either encourage adequate fluid intake to ensure euhydration or measure hydration status and incorporate as a covariate into the analysis.

The differences in FM and FFM between DXA and IB770 underscore the need for caution when interpreting body composition data depending on the method of assessment. Despite these discrepancies, the IB770 has shown reliability for detecting the relative change in body composition throughout a hypocaloric dietary intervention. [37]. Additionally, other studies have shown agreement between IB770 and DXA for FFM changes during a diet and exercise intervention in older adults with type 2 diabetes [45] and cross-sectionally in adults from multiple BMI categories [46]. Thus, if a researcher or clinician is more concerned with precision of measurement, the DXA may be preferable as it is more robust to pretesting standardization error and differentiates between the lean soft tissue and bone mineral content of fat-free mass [47]. However, IB770 may perform just as well for assessing relative changes in FM and FFM over time.

We did not assess the menstrual cycle in our study, and 59% of our participants did not use contraceptives. Some research has identified an increase in extracellular fluid retention during menstruation [48], whereas others have observed consistency of DXA-derived body composition estimates across the menstrual cycle [49]. As part of a 4C model, Gould et al. reported greater body volume, extracellular fluid, and body mass, though these measures did not exceed measurement error. Given that the purpose of our investigation was to assess the validity of IB770 measures from a single timepoint rather than across the menstrual cycle, we believe the expected variability in sex hormone concentration between our participants increases our confidence in the robustness of the agreement between methods.

Methodological differences in the pre-visit conditions may also explain differences in our study compared with previous observations. We standardized food and fluid intake in our study, with participants consuming nothing by mouth 10–12 h prior to the laboratory visit. Future investigations should continue either to standardize or to control for fluid consumed in the hours prior to measurement, particularly for studies with repeated measures. Our findings may be expanded upon by collecting measures of hydration status (i.e., 24 h urine osmolality, 24 h urine specific gravity, or plasma osmolality) to determine TBW content when participants are euhydrated. However, TBW content among our participants was likely to be stable since they did not exercise prior to testing and therefore were unlikely to experience acute fluid loss. In fluid homeostasis of body systems, a minor TBW deficit would be offset by an increase in arginine vasopressin to conserve fluid within the kidneys and thereby restore fluid balance [50]. Furthermore, a previous investigation found the difference between IB770 and DXA was not influenced by hydration status assessed via urine specific gravity [51]. Last, we appeared underpowered to detect the pre-specified equivalence bounds that we deemed most clinically relevant. After completion of this study, a retrospective power analysis was conducted using the “blandPowerCurve” function from the “SimplyAgree” package in R, based on the methods of Lu et al. [52]. This power analysis suggested based on our observed data, we could detect with 80% power a 7.1 kg equivalence for FFM with 48 participants, a 4.3 kg equivalence for FM with 51 participants, a 4% equivalence for body fat percentage, and a 3.5 L equivalence for TBW. A sensitivity analysis with these adjusted regions demonstrated consistency of agreement with the wider equivalence margins. Thus, results from our equivalence test, while promising, serve to encourage a larger verification of the consistency of these measures if a smaller equivalence region is required in clinical practice or the pooling of our results in a meta-analysis.

## 5. Conclusions

Our results suggest the IB770 is a valid, low-cost alternative for assessing total body water in a racially diverse sample of female participants compared with the deuterium oxide dilution method. This specific device showed greater consistency with reference criteria compared with the similar multifrequency device from another investigation, albeit with a different choice of reference criteria. However, users should be aware that IB770 may overestimate FFM and may provide more error when assessing FM in females with higher levels of body fat compared with DXA. For the best precision in body composition assessment, we recommend continued use of DXA to obtain bone mineral density and/or body volume, while TBW may be obtained from IB770. A 4C model using total body water derived from IB770 may be used interchangeably with the deuterium dilution method for assessing total body water.

## Figures and Tables

**Table 1:** Comparison of means and standard deviations for the four-compartment model using TBW derived from D2O or IB770.

	4C Model with D2O	4C Model with IB770
**Fat Mass (kg)**	22.28 ± 13.24	22.04 ± 13.26
**Fat-Free Mass (kg)**	46.63 ± 6.71	46.87 ± 6.66
**Body Fat %**	29.87 ± 12.16	29.47 ± 12.37
**TBW (L)**	31.89 ± 5.91	32.24 ± 5.87
**FFM Hydration**	0.68 ± 0.05	0.68 ± 0.04

## Data Availability

Please contact the corresponding author (kingra14@kennesaw.edu) for inquiries regarding data availability.

## Abbreviations

The following abbreviations are used in this manuscript:

IB770	Inbody 770
D2O	Deuterium oxide
TBW	Total body water
4C	Four-compartment model
CCC	Lin’s concordance correlation coefficient
FM	Fat mass
FFM	Fat-free mass
BIA	Bioelectrical impedance
DXA	Dual-energy X-ray absorptiometry
BV	Body Volume
BMC	Bone mineral content
